# Investigating the Impact of Resident Doctor Regional Research Meetings on Research Outcomes: A 20-Year Longitudinal Analysis of the Regional Bardhan Fellowship Day

**DOI:** 10.7759/cureus.78839

**Published:** 2025-02-11

**Authors:** Arkadeep Dhali, Olufunmilola Oni, Mohamed G Shiha, Suneil A Raju, Flora Kokwaro, Andrew Nelson, Matthew Huggett, Sampath Kumar, David S Sanders

**Affiliations:** 1 Department of Gastroenterology, Sheffield Teaching Hospitals NHS Foundation Trust, Sheffield, GBR; 2 School of Medicine and Population Health, University of Sheffield, Sheffield, GBR; 3 Department of Gastroenterology, Hull University Teaching Hospitals NHS Trust, Hull, GBR; 4 Department of Gastroenterology, Leeds Teaching Hospitals NHS Trust, Leeds, GBR; 5 Department of Gastroenterology and Hepatology, Doncaster Royal Infirmary, Doncaster, GBR

**Keywords:** academia, curriculum, gastroenterology, regional teaching, research fellowship

## Abstract

Introduction

Medical research plays a critical role in advancing medical knowledge and improving patient care. However, recent studies indicate a decline in trainee participation in research activities. The Yorkshire and Humber regional trainee Bardhan Fellowship was established to address this issue by encouraging and motivating gastroenterology trainees to engage in research. This study aims to evaluate the outcomes of abstracts presented at this regional annual gastroenterology conference.

Methods

Over the past 20 years, data collection has been conducted using three primary methods. A questionnaire was distributed to gather feedback and ratings from the participants. Additionally, final meeting programs were analyzed to identify abstracts and their respective presenters, including those ranked in the top three. A cross-referencing approach was employed to track subsequent publications of presented abstracts using Web of Science and Medical Literature Analysis and Retrieval System Online (MEDLINE) databases.

Results

An analysis of 259 abstracts revealed that 91 (35%) were published as full papers in peer-reviewed journals, whereas 168 (65%) remained unpublished. Of the 67 top-three ranked abstracts, 38 (57%) achieved full publication, a significantly higher rate than the 52 (27%) among the 192 unranked abstracts (p<0.0001). The median time to publication for the 67 ranked abstracts was 12.5 months (interquartile range {IQR}: 6.25-21.25 months), compared to 15 months (IQR: 6-27 months) for the 192 unranked abstracts. Ranked abstracts were published in journals with a median impact factor of 3.769 (IQR: 2.491-7.527; p<0.0001), while unranked abstracts were published in journals with a median impact factor of 2.884 (IQR: 1.95-4.628; p<0.0001). Ranked presenters were more likely to receive higher research degrees (26/39 {67%} versus 33/73 {45%}, p=0.03) and were more likely to be employed in a university tertiary care setting (28/41 {68%} versus 34/77 {44%}, p=0.02). Questionnaire data from 161 attendees over nine years indicated a positive evaluation of the meeting.

Conclusion

Research training conferences are well received by resident doctors and help them develop their research and presentation skills. This conference model could be implemented in other regions to promote research dissemination among resident doctors.

## Introduction

Medical research is a cornerstone in the continuous evolution of medical knowledge, serving as a foundation for advancements across the field. When paired with professional society meetings, research becomes a driving force for further progress by fostering collaboration and the exchange of ideas among medical professionals. These meetings provide a crucial platform for trainees to present their research in abstract form, allowing them to receive constructive feedback from peers, which often helps refine their work for eventual publication.

Sanders et al. have previously shown that 69.8% of abstracts from a single British Society of Gastroenterology meeting were successfully published in full [1]. More recently, a study by Raju et al. revealed that 30.9% of abstracts presented at the United European Gastroenterology Week were published, confirming a continued downward trend in research dissemination [2].

Furthermore, only 55.6% of gastroenterology specialist trainees feel confident that they will be able to develop the required expertise in their sub-specialist area [3]. Since the implementation of the new Shape of Training curriculum, providing training time for research has become more difficult, and it is recognized that more research time should be built in [3]. Therefore, there is a need for training opportunities to develop research skills for specialist trainees. In response to this apparent decline in gastroenterology research productivity and to support gastroenterology specialty trainees, the Bardhan Fellowship was established [4]. Initiated in 2003 in South Yorkshire, this annual event invites gastroenterology trainees from Yorkshire and Humber to present their research, with the aim of fostering and recognizing their contributions to the field. It is a way of encouraging people to consider research and then give them a platform to present their work in a less stressful and low-cost environment in a peer-to-peer process. Currently, this fellowship is recognized in the regional gastroenterology higher specialty training program in the Yorkshire and Humber deanery. The fellowship (www.sheffieldgastro.nhs.uk/the-bardhan-fellowship/) is named in honor of Professor Bardhan, known as the "grandfather" of gastroenterology in South Yorkshire and recipient of the first-ever lifetime achievement award from the British Society of Gastroenterology, and serves as a tribute to his mentorship and leadership in the discipline.

The objective of this study is to evaluate the outcomes of abstracts presented at the annual Bardhan Fellowship meetings, with a focus on determining the role of the event. Success will be measured by the proportion of abstracts that are subsequently published as full articles in peer-reviewed journals, the proportion of presenters who have achieved a higher doctoral research degree (MD/PhD), and the proportion of them employed at tertiary care university hospital settings. In addition, feedback from meeting attendees will be analyzed to assess the broader impact and value of the fellowship in promoting gastroenterology research.

The article's abstract was presented at the Royal College of Physicians Med+ 2024 Conference in London.

## Materials and methods

Bardhan Fellowship program

During the Bardhan Fellowship meetings, all participants who submit abstracts for this program are given a chance to present, and there is no submission or registration fee. Each speaker (20-40 participants each year) is allocated eight minutes to present their research, followed by a two-minute question-and-answer session with the audience. Presentations are peer-reviewed, with the top three presenters being awarded based on scores (using a first, second, and third ranking with no other rankings beyond this) in three key areas: originality of the work, presentation skills, and the ability to address questions effectively. Attendees (consultants and specialty registrars) are provided with a program that allows them to score each presentation and leave feedback. These scores are then collected, tallied, and used to determine the overall ranking of presenters. Scores attributed are A=three points, B=two points, and C=one point. The individual with the highest score is declared the winner and awarded a monetary prize (£500).

Questionnaire

At the conclusion of each meeting, an optional questionnaire was distributed to all attendees to gather feedback on the event. The questionnaire was divided into five sections. In the first section, attendees rated their overall experience, with options ranging from "very poor" to "excellent." The second section asked attendees to express their agreement or disagreement with specific aspects of the meeting, using a binary agree/disagree scale. The third section invited opinions on the live endoscopy sessions and the special Prize sessions. In the fourth section, the participants compared the Bardhan Fellowship to other regional meetings in Yorkshire. The questionnaire concluded with an optional free-text field for suggestions and comments. The structured questions provided quantifiable data for analysis, while the open-text responses yielded qualitative insights. Responses collected were compiled into an Excel sheet for analysis.

Search methods

From 2003 to 2023, 249 abstracts were presented at the Bardhan Fellowship meetings. These abstracts were systematically tracked by two authors through two databases, Web of Science and Medical Literature Analysis and Retrieval System Online (MEDLINE), to determine if they had been published as full articles. Searches were conducted using keywords from the abstract titles and the names of the first authors, as recorded in the final Bardhan Fellowship programs. Once a potential full article was identified, it was cross-referenced with the corresponding abstract to confirm that it represented the same study. Data on published abstracts were entered into an Excel sheet, with published studies highlighted in green. Additionally, the publication month, journal name, and time interval from presentation to publication were recorded. The impact factors of the journals (at the time of publication), sourced through Google searches, were also added to the Excel sheet for further analysis.

Data analysis

For comparative purposes, percentages were calculated for categorical data. Median and interquartile range (IQR) values were computed for continuous variables. Categorical variables were compared using the chi-square test, while the Mann-Whitney U test was employed to compare continuous variables between ranked and unranked groups. Correlation tests were also performed where necessary. A p-value of <0.05 was considered statistically significant. All statistical analyses were performed using GraphPad Prism 10.0.1 (GraphPad Software Inc., La Jolla, CA).

## Results

An analysis of 249 abstracts revealed that 88 (35%) were subsequently published as full papers in peer-reviewed journals, while the remaining 161 (65%) remained unpublished in abstract form. Of the 67 (some years had joint top three rank holders) abstracts ranked in the top three at their respective presentations, 38 (57%) achieved full publication, demonstrating a significantly higher likelihood of publication compared to the 50/182 (27%) publication rate among unranked abstracts (p<0.0001). Ranked abstracts accounted for 38/88 (43%) of all published abstracts, while 50/88 (57%) were from unranked categories.

**Figure 1:**
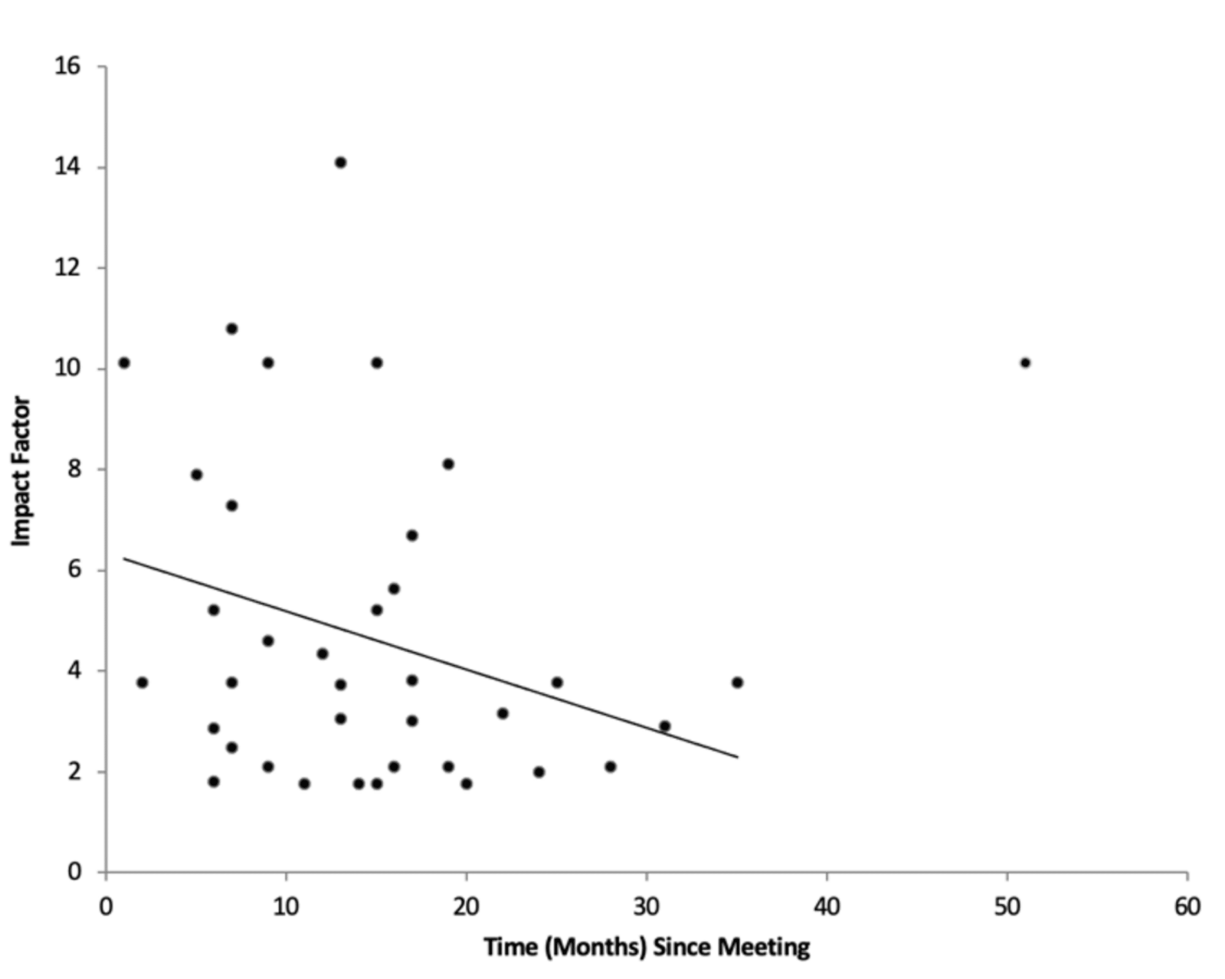
The relationship between impact factor and lag time.

**Figure 2:**
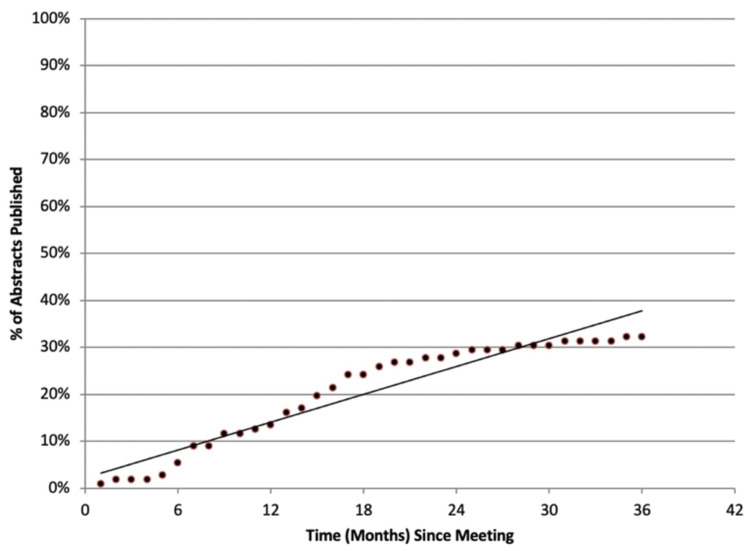
The relationship between lag time and the percentage of published abstracts.

The median time to publication (lag time) for the 182 unranked abstracts was 15 months (IQR: 6-27 months), compared to 12.5 months (IQR: 6.25-21.25 months) for the 67 ranked abstracts. The overall median lag time for both groups combined was 13 months (IQR: 6-25 months). Notably, longer lag times were associated with lower journal impact factors. The median impact factor for journals publishing ranked abstracts was 3.769 (IQR: 2.491-7.527; p<0.0001), while the median impact factor for journals publishing unranked abstracts was 2.884 (IQR: 1.95-4.628; p<0.0001). A weak negative correlation was observed between the time elapsed since the meeting and the journal impact factor (Figure 1). Conversely, a positive correlation was found between the number of abstracts published and the time since the meeting (Figure 2). On average, four abstracts (IQR: 3-5) per year were published from each meeting.

A total of 249 abstracts were presented by 150 presenters. Of these 150 presenters, 119 (79%) were resident doctors (foundation doctors, senior house officers, and registrars), 28 (19%) were medical students, two (1%) were dietitians, and one (0.6%) was a gastrointestinal physiologist. Among the 119 resident doctors, 59 (50%) held a higher research degree (MD/PhD), and 62 (52%) were employed at a university tertiary care setting. Ranked presenters were more likely to have a higher research degree (26/39 {67%} versus 33/73 {45%}; OR=2.43; p=0.03) and were more likely to be employed at a university tertiary care setting (28/41 {68%} versus 34/77 {44%}; OR=2.65; p=0.02).

Questionnaire data were collected from 161 attendees, with 145/161 (90%) rating the overall meeting experience as excellent and 16/161 (10%) rating it as good. The event was widely regarded as an "excellent platform for presenting completed research with constructive feedback and praise." The participants particularly appreciated the live endoscopy sessions and felt that there was sufficient time for meaningful discussion.

## Discussion

In this retrospective analysis of our prospectively collected data over 20 years, we found that approximately one in three abstracts presented at the Bardhan Fellowship was successfully published in peer-reviewed journals. Trainees whose work ranked in the top three at their respective meetings were significantly more likely to publish their research (57% versus 27%, p<0.0001) and were more likely to publish in journals with a higher impact factor (p=0.02). Although the difference in time to publication was not statistically significant (p=0.54), ranked abstracts generally reached publication in less time than non-ranked ones. Based on these findings, we recommend incorporating a competitive peer-review process into similar initiatives to foster higher-quality research outputs.

Our results align with findings from previous studies and conferences regarding publication rates. Scherer et al. reported a publication rate of 37.3%, slightly higher than our rate, and found that abstracts accepted for presentation at meetings were more likely to be published, a finding that supports the value of the Bardhan Fellowship in providing a platform for work to be shared [5]. Similarly, Gandhi et al. found a publication rate of 31.5%, consistent with our results, although they concluded that winning awards at their meetings did not correlate with publication, contrasting with our finding that a top three ranking significantly increased the likelihood of full publication [6]. In terms of lag time, our results are comparable to those of Raju et al., who reported a median lag time of 15 months, the same as in our study [2]. Their publication rate of 30.9% was also in line with our findings.

Our data indicate that the number of abstracts published annually has remained stable (an average of 13 abstracts per year), countering the trend of declining research output reported by Hopper et al. [4]. Nevertheless, various factors, including limited mentorship, publication bias, and inadequate resources, can hinder the progression of abstracts to full publication [7,8]. Feedback from attendees further underscores the effectiveness of the meeting, with most participants deeming it highly educational. Additionally, the increased enthusiasm for research among attendees aligns with the original purpose of the Bardhan Fellowship and highlights its ongoing success. Indeed, studies have shown that residents' active participation in research improves their skills and accelerates career development [9,10], although persistent barriers, such as insufficient time or funding, remain [11,12].

The 2022 annual census of the Royal College of Physicians (RCP) revealed that while 40% of respondents were involved in research, 60% were not, with those in acute or general internal medicine being less likely to participate [13]. The RCP emphasized the importance of addressing these challenges to support early-career clinicians in research and ensure that the NHS can drive future innovation. In line with this, the Royal Society of Medicine's (RSM) mission to promote learning and support innovation highlights the value of research training days [14]. The Bardhan Fellowship regional training day at the Yorkshire and Humber deanery could serve as a pilot initiative for expansion into other deaneries and, more so, across various specialty programs nationwide.

Expanding such training opportunities would help address current disparities in research access and strengthen clinical expertise. Structured training and mentorship programs have been identified as key strategies to encourage academic productivity among medical trainees [15,16].

This study has several notable strengths. First, the use of a cross-referencing method ensured that all potential publications from the presented data were captured, making our results highly accurate and reflective of true publication rates. Second, the prospective collection of data over 20 years, combined with information from multiple sources, allowed us to identify trends and provide a comprehensive analysis. This is the first evaluation of a regional gastroenterology research meeting.

Nevertheless, there are certain limitations. Although we found that 50% of authors and presenters hold advanced degrees, some may have presented projects stemming from their degree work. Additionally, feedback data were unavailable for several years, which may have restricted the number of responses. Additionally, because the questionnaire was administered at the end of the meeting, those who left early missed the opportunity to provide feedback. The meeting may also have been insufficiently advertised across Yorkshire, limiting participation by all trainees. Furthermore, as our study period predates the median lag time for abstracts presented in 2023, the full publication outcomes of these recent abstracts are yet to be realized. Also, it is difficult to measure the direct benefit of the regional research meeting on subsequent publications. However, resident doctors have commented on the value of the feedback given, and it is likely that this has supported subsequent manuscript writing.

## Conclusions

In summary, our study highlights the positive reception of the Bardhan Fellowship since its launch, showcasing its substantial benefits for both presenters and attendees. The program keeps participants updated on advancements in the field while providing a supportive setting for public speaking practice. With a publication rate of 35%, the Bardhan Fellowship serves as a successful model that could be adapted in other regions to foster academic growth and research dissemination.
